# Carbonized Cotton Fabric-Based Flexible Capacitive Pressure Sensor Using a Porous Dielectric Layer with Tilted Air Gaps

**DOI:** 10.3390/s21113895

**Published:** 2021-06-04

**Authors:** Yelin Ko, Chi Cuong Vu, Jooyong Kim

**Affiliations:** Department of Organic Materials and Fiber Engineering, Soongsil University, Seoul 06978, Korea; yelinko@ssu.ac.kr (Y.K.); vuchicuong@soongsil.ac.kr (C.C.V.)

**Keywords:** capacitive pressure sensor, carbonized cotton fabric (CCF), carbonization, porous dielectric layer, particle–template method, tilted air gaps

## Abstract

Flexible and wearable pressure sensors have attracted significant attention owing to their roles in healthcare monitoring and human–machine interfaces. In this study, we introduce a wide-range, highly sensitive, stable, reversible, and biocompatible pressure sensor based on a porous Ecoflex with tilted air-gap-structured and carbonized cotton fabric (CCF) electrodes. The knitted structure of electrodes demonstrated the effectiveness of the proposed sensor in enhancing the pressure-sensing performance in comparison to a woven structure due to the inherent properties of naturally generated space. In addition, the presence of tilted air gaps in the porous elastomer provided high deformability, thereby significantly improving the sensor sensitivity compared to other dielectric structures that have no or vertical air gaps. The combination of knitted CCF electrodes and the porous dielectric with tilted air gaps achieved a sensitivity of 24.5 × 10^−3^ kPa^−1^ at 100 kPa, along with a wide detection range (1 MPa). It is also noteworthy that this novel method is low-cost, facile, scalable, and ecofriendly. Finally, the proposed sensor integrated into a smart glove detected human motions of grasping water cups, thus demonstrating its potential applications in wearable electronics.

## 1. Introduction

Flexible pressure sensors have received tremendous attention due to their potential in monitoring human health [1,2,3], human–machine interaction systems [4,5], and intelligent robotics [6,7,8]. Thus far, numerous strategies have been proposed to develop pressure sensors with excellent sensing performance based on various sensing mechanisms, including piezoresistive [9,10], capacitive [11,12,13,14], piezoelectric [15,16], and triboelectric effects [17]. Among these approaches, a capacitive-type pressure sensor has attracted the most interest owing to its fast response, high reversibility, temperature insensitivity, simple structure and fabrication process, and low power consumption [18,19]. In general, a capacitive pressure sensor consists of two parallel electrodes and a dielectric layer placed between them. When pressure is applied to a capacitive-type sensor, the thickness of the dielectric decreases, thus changing the capacitance variation in the sensor.

However, capacitive pressure sensors typically exhibit low sensitivities due to the relatively small changes in the capacitance of parallel plates [20]. Therefore, several methodologies have been developed to tune the performance of capacitive pressure sensors, which are mostly determined by the deformability of the dielectric layer. One of the most popular efforts to modify the dielectric layer for sensitivity enhancement involves engineering microstructures on the elastomer surface. Microstructured patterns, such as convex [21], pyramids [22], pillars [23], and waves [24], have been proved to be effective in yielding ultrahigh sensitivity. However, the working range is typically limited to <10 kPa because the patterns quickly collapse even when subjected to low pressure, thereby making the strategy less desirable for most application settings [1]. In addition, this method requires time-consuming, expensive, and complicated process steps to fabricate the microstructure silicone mold.

Another approach that focuses on the formation of porous structures inside the dielectric layer has been proposed as an alternative to the microstructuring of elastomers, including various fabrication techniques such as the particle–template method [1,11,25,26], chemical foaming method [27], and emulsion–template method [28]. Among these, the particle–template method has emerged as a promising strategy with great potential for providing higher deformability to resulting sensors, along with ease of fabrication at low cost. In this strategy, easily dissolvable particles (sugar or NaCl) are added to uncured silicone rubber, such as polydimethylsiloxane (PDMS) [1,11] or Ecoflex [25,26]. After the silicone elastomers are cured, the residual particles are dissolved and removed to obtain a porous dielectric layer. In addition, this method can offer a wide dynamic working range to resulting sensors. In general, it has been known that typical tactile pressures are distributed in low-pressure regimes (gentle touch, 0−10 kPa) and medium-pressure regimes (object manipulation, 10−100 kPa) [20,29]. Therefore, in order to monitor human motions, a pressure sensor needs to be sensitive over a working range up to 100 kPa [26] to cover the overall tactile pressures. It has been frequently reported that pressure sensors fabricated by the particle–template strategy can work at the low-pressure (0−10 kPa) [25] and medium-pressure regimes (10−100 kPa) [11,12] and broader ranges (130 kPa [26], 400 kPa [1]).

The structured pores inside elastomers have been demonstrated to be effective in improving the sensitivity of capacitive pressure sensors by reducing the stiffness of elastomers and increasing the effective dielectric constant due to the gradual closure of pores. For example, Hwang et al. [1] demonstrated that a hierarchically structured porous PDMS-based pressure sensor exhibits 22.5 times greater sensitivity (1.8 × 10^−1^ kPa^−1^) than a bulk PDMS-based sensor. Atalay et al. [11] proposed a pressure sensor using porous PDMS and textile electrodes with an improved sensitivity of 1.21 × 10^−2^ kPa^−1^. Kwon et al. [26] fabricated a sensor with porous Ecoflex and showed that the sensitivity of the resulting sensor was 37.6 times higher than that of a sensor with a bulk dielectric structure. However, the microporous structure still experiences sensitivity saturation in high-pressure regions [14,30]. In addition, the high porosity of the dielectric layer may affect the mechanical stability of the sensor [31] and generate more noise.

Meanwhile, the design of electrodes of a capacitive pressure sensor has been less considered owing to its less apparent relationships with sensitivity. Electrodes are mainly fabricated by mixing conductive nanomaterials, such as Ag nanowires [13,19], graphene [18], and carbon nanotubes [12,30], with flexible substrates or polymer matrices [21]. These nanomaterials are widely known to possess excellent mechanical and electrical properties. However, contact with these materials may pose health risks to manufacturers and users, which should not be neglected for use in human health monitoring. In addition, they require complicated fabrication processes and expensive raw materials. Carbonized cotton fabric (CCF), which is converted from cotton fabrics by a simple thermal treatment, can be a potential candidate to compensate for these drawbacks. The high-temperature carbonization process in low oxygen concentration leaves a carbon skeleton formed from cellulose fibers, and the biomass-derived carbon-based materials exhibit high electrical conductivity and flexibility [32]. Furthermore, the carbonization process is scalable, low-cost, and ecofriendly.

Despite their potential, to the best of our knowledge, CCF has rarely been used as an electrode in capacitive pressure sensors even though some studies have highlighted its application to supercapacitor electrodes [33], strain sensors [34,35], and resistive-type pressure sensors [32,36]. With efforts to enhance the sensitivity by engineering the dielectric layer, a CCF-based capacitive pressure sensor may be widely utilized in a variety of applications taking advantages of its less hazard to the human body and general benefits of a capacitive-type sensor. Herein, we propose a facile and inexpensive yet effective strategy to fabricate a capacitive pressure sensor based on CCF electrodes and a porous Ecoflex as the dielectric layer. We optimized the sensor performance in two steps: (1) exploring the effects of textile structures (woven vs. knitted) of CCF on sensitivity and (2) structuring the porous elastomer by introducing air gaps (vertical vs. tilted) to generate additional voids inside. Our sensor, consisting of a porous Ecoflex with tilted air gaps and knitted CCF electrodes, exhibited high sensitivity, which can be attributed to the enhanced deformability of the dielectric and the rough and bulky nature of the knitted structure of electrodes. In addition, the sensor showed delayed pressure-sensing saturation, wide working range, low hysteresis, and high reversibility and durability. As a practical demonstration, we integrated the proposed sensor into a smart glove to monitor human motions during object grasping.

## 2. Materials and Methods

**Figure 1:**
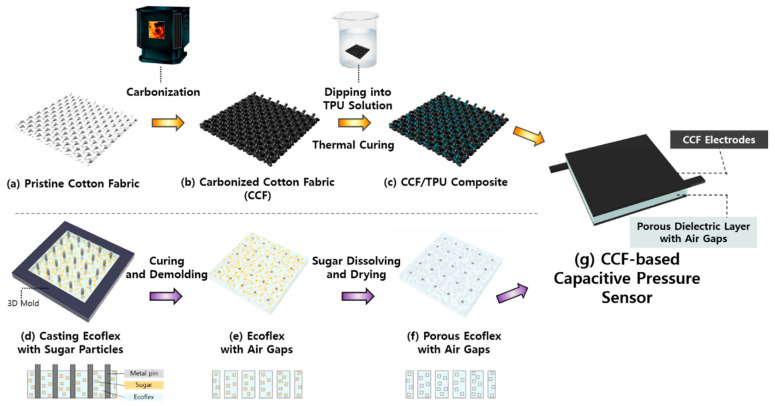
Manufacturing process of carbonized cotton fabric (CCF)-based capacitive pressure sensors: (**a**–**c**) fabrication of CCF electrodes; (**d**–**f**) fabrication of a porous dielectric layer with air gaps; (**g**) developed capacitive pressure sensor using CCF electrodes and a porous dielectric layer with air gaps.

To prepare the electrodes of the capacitive pressure sensor, common 100% cotton fabrics (woven: 181.3 g·m^−2^, 0.31 mm in thickness; knitted: 154.8 g·m^−2^, 0.32 mm in thickness) (Figure 1a) were put in an aluminum container and placed in a furnace (B253-DK, Hanwon, Korea) for subsequent carbonization treatment. The carbonization process of these pristine cotton fabrics lasted for 1 h after reaching 800 °C, starting from room temperature. After naturally cooling to room temperature, the CCF (Figure 1b) was rinsed several times with distilled water and completely dried to remove impurities and ashes from its surface. Since CCF is too fragile to be used in its original state, we used thermoplastic polyurethane (TPU) as a supporting substrate, similar to previous studies [32,37] to improve the mechanical strength. A 7 wt % TPU solution was obtained by dissolving TPU in N,N-dimethylformamide (DMF) with magnetic stirring at 50 °C and 200 rpm for 6 h. To prepare the CCF/TPU composite with moderate flexibility and strength (Figure 1c), the CCF was dipped in the TPU solution for 5 min and dried at 80 °C for 5 min; this process was repeated three times before the final thermal curing at 80 °C for 120 min. Finally, the CCF/TPU composite material was cut into a size of 1.4 cm × 1.4 cm with a small extra tip for wire connection (0.6 cm × 0.2 cm) using a laser cutting machine.

A porous dielectric layer with air gaps was prepared by casting Ecoflex with sugar particles (Figure 1d). The Ecoflex (Shore 00-30 hardness, Smooth-on Inc., Macungie, PA, USA) prepolymer solution was obtained by mixing a base (Part A) and cured agent (Part B) at a weight ratio of 1:1. Commercial granulated brown sugar was integrated into the solution at a ratio of 1:2.5 by weight. The Ecoflex solution with sugar granules was poured into a 3D mold with a length, width, and height of 1.4, 1.4, and 0.4 cm, respectively. With a 5 × 5 array of metal pins (0.5 mm length × 0.5 mm width per pin) inserted into the solution, it was instantly cured at 150 °C for 3 min to prevent layer separation in Ecoflex and sugar particles. When structuring tilted air gaps in Ecoflex, metal pins were inserted at a tilting angle of 60°. Subsequently, the solution was placed in an oven at 80 °C for 2 h for further thermal curing. After the pins were removed, the cured Ecoflex with sugar particles was removed from the 3D mold (Figure 1e). A porous dielectric elastomer with air gaps was prepared (Figure 1f) after dissolving the sugar particles in distilled water with magnetic stirring at 70 °C and 120 rpm for at least 24 h. Finally, the capacitive pressure sensor was obtained by sandwiching the air-gapped porous dielectric layer between the CCF electrodes (Figure 1g). To minimize any adverse effects of additional adhesive layers on the performance of the sensor, we hand-sewed the three layers using a common needle and thread.

## 3. Results and Discussion

### 3.1. Structures of the Sensor

**Figure 2:**
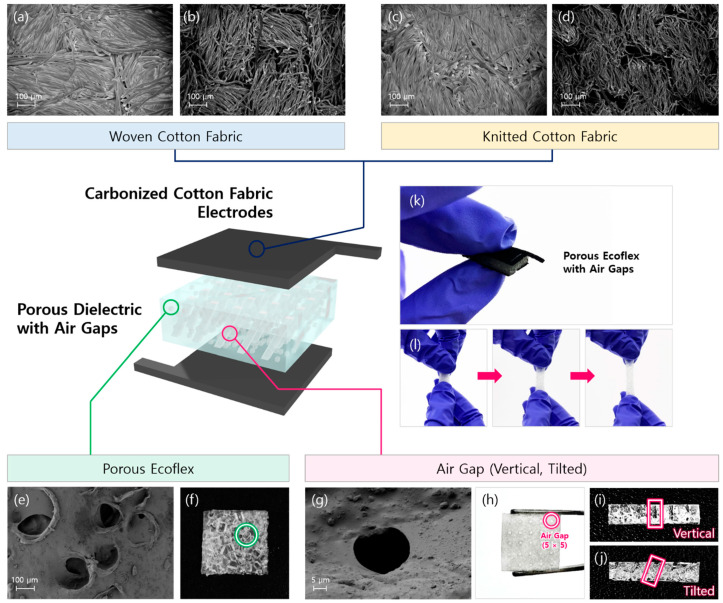
Structure of the CCF-based capacitive pressure sensor and SEM images of the electrodes and dielectric layer. (**a**) Woven pristine cotton fabric; (**b**) woven CCF; (**c**) knitted pristine cotton fabric; (**d**) knitted CCF; (**e**) porous structure of Ecoflex as a dielectric layer; (**f**) top-view image of the porous Ecoflex. The voids inside Ecoflex are the pores created by sugar particles and highlighted in green; (**g**,**h**) air gaps in the porous dielectric; cross-sectional view images of the vertical (**i**) and tilted air gaps (**j**) in the porous dielectric. The air gaps were formed by metal pins and highlighted in pink; (**k**) real image of the CCF-based capacitive pressure sensor with air gaps; (**l**) high stretchability of the dielectric layer.

The surface morphology of the CCF-based capacitive pressure sensor was obtained by scanning electron microscopy (SEM) (GeminiSEM 300, Carl Zeiss, Oberkochen, Germany). The proposed sensor consists of an air-gapped porous dielectric layer sandwiched between flexible CCF electrodes. In this study, the woven and knitted textile structures of electrodes were first compared to optimize the sensing performance (Figure 2a–d). The fibers in carbonized fabric became finer than those in pristine cotton fabric, indicating that carbon-based materials remained after the carbonization process. The inherent structures of the woven (Figure 2a,b) and knitted structures (Figure 2c,d) were retained in carbonized fabric. Figure 2e,f presents the porous structure of the dielectric layer formed by mixing the Ecoflex solution with sugar particles. Figure 2g,h shows the air gaps formed by metal pins that created additional voids during thermal curing. It is evident from cross-sectional images that vertical (Figure 2i) and tilted air gaps (Figure 2j) were clearly observed. Figure 2k shows the real images of the capacitive pressure sensor with the CCF electrodes and porous dielectric with air gaps. As presented in Figure 2l, the Ecoflex dielectric layer was highly stretchable.

### 3.2. Sensing Mechanism

**Figure 3:**
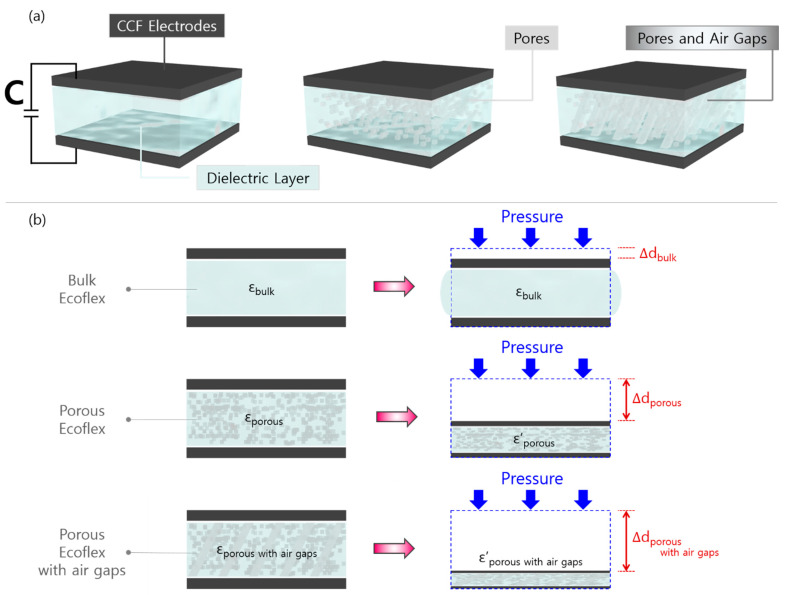
Pressure-sensing mechanisms of capacitive pressure sensors using a bulk Ecoflex, porous Ecoflex, and air gapped-porous Ecoflex. (**a**) Schematic of sensors with three different structures in the dielectric; (**b**) schematic of the structural deformation of capacitive pressure sensors using a bulk, porous, air gapped-porous Ecoflex dielectric layer during compressive loading.

Figure 3 describes the working principle of the proposed capacitive sensor based on a porous dielectric layer with air gaps and CCF electrodes. In general, the capacitance of a capacitive pressure sensor (C_Sensor_) (Figure 3a) can be calculated as follows (Equation (1)):

(1) $$C_{\mathrm{Sensor}}=\varepsilon_{0}\varepsilon_{r}\frac{A}{d_{0}}$$

where $\varepsilon_{0}$ is the dielectric permittivity of vacuum, $\varepsilon_{r}$ is the dielectric permittivity of dielectric material, A represents the area of overlapped electrodes, and $d_{0}$ represents the thickness of the dielectric layer. Therefore, the capacitance variation in a sensor under applied pressure is determined by the changes in thickness and the relative permittivity of the porous Ecoflex.

Figure 3b presents CCF-based capacitive pressure sensors in the initial and loading states under the same pressure level. When pressure is applied to a pressure sensor with a bulk dielectric without pores, it experiences the barreling phenomenon [26]. As the dielectric constant of the bulk Ecoflex $(\varepsilon_{bulk}$) remains the same under compression, the capacitance variation in the sensor largely depends on the changes in distance between the electrodes (Δd_bulk_). Meanwhile, the pores integrated into a solid silicone elastomer can have two benefits, which can significantly improve the sensitivity of CCF-based pressure sensors. First, the porous dielectric is subjected to larger deformation compared to the bulk dielectric under the same level of pressure owing to numerous pores. This leads to greater changes in the thickness of the porous Ecoflex (Δd_porous_), which results in greater variations in the capacitance responses of the sensor.

Second, the pores under external pressure increase the effective dielectric constant of the porous *Ecoflex* ($\varepsilon_{e}$), which can be described as follows (Equation (2)) [11]:

(2) $$\varepsilon_{e}=\varepsilon_{air}V_{air}+\varepsilon_{Ecoflex}V_{Ecoflex}$$

where $\varepsilon_{air}$ = 1 and $\varepsilon_{Ecoflex}$ = 2.8 [38], *V_air_* represents the volume fraction of air, and *V_Ecoflex_* represents the volume fraction of pristine Ecoflex. Under compression, the pores in the dielectric layer steadily close, decreasing the volume fraction of air and increasing that of Ecoflex. The lower dielectric constant of pores ($\varepsilon_{air}$ = 1) is replaced by the higher dielectric constant of silicone elastomer ($\varepsilon_{Ecoflex}=$ 2.8), which subsequently increases the effective dielectric constant of the porous Ecoflex composite [1]. When air gaps are introduced to this porous elastomer, the sensitivity of the pressure sensor can be further improved owing to enhanced deformability because the gaps will reduce the stiffness of the pristine Ecoflex, creating additional voids inside.

### 3.3. Sensitivity Differences between Pressure Sensors Based on Woven and Knitted CCF Electrodes

**Figure 4:**
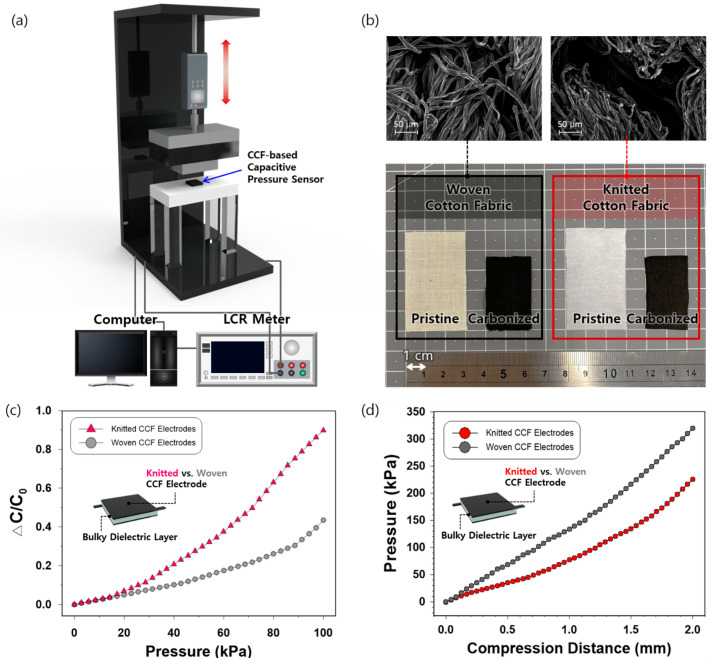
(**a**) Schematic of the customized universal testing machine; (**b**) woven and knitted cotton fabrics after carbonization at 800 °C; (**c**) pressure–response curves of capacitive pressure sensors with knitted and woven CCF electrodes; (**d**) relationship between the compression distance and applied pressure of sensors with knitted and woven CCF electrodes.

We optimized the sensitivity of the CCF-based capacitive pressure sensor with the following two steps: (1) investigation of the effects of textile structures (woven vs. knitted) of CCF electrodes and (2) exploration of the effects of vertical and tilted air-gap structures of the porous dielectric layer. The pressure-sensing performance of the sensor was evaluated using a customized universal testing machine (Dacell Co., Seoul, Korea) and a Keysight E4980AL LCR meter (Figure 4a). Figure 4b shows photographs of pristine and carbonized woven and knitted cotton. After the carbonization process at 800 °C, the colors of both pristine woven and knitted cotton changed to black, indicating that the carbon materials were mainly left after the high-temperature pyrolysis. The surface area and weight of the pristine cotton fabrics were reduced by 49.6% and 89.3% for the woven structure and 47.2% and 90.5% for the knitted structure, respectively. The thickness of both woven and knitted CCF decreased from 0.31 to 0.25 mm and 0.32 to 0.25 mm, respectively. As shown by the SEM images, naturally formed spaces were observed in the knitted CCF owing to its inherent structure, whereas such space was less present in the woven CCF (Figure 4b).

To compare the pressure-sensing sensitivity caused by the two different structures of CCF electrodes, the woven and knitted CCF electrodes were combined with a bulk Ecoflex as a dielectric layer without pores and air gaps. The sensitivity of CCF-based capacitive pressure sensors (S) was calculated as S = △C/C_0_/P, where △C and C_0_ represent the change in capacitance and initial capacitance of the sensor, respectively, and P represents the applied pressure. As shown in Figure 4c, the pressure sensor with knitted CCF electrodes exhibited higher sensitivity (9.0 × 10^−3^ kPa^−1^) than the woven structure (4.4 × 10^−3^ kPa^−1^) under an applied pressure level of 100 kPa. The sensitivity difference between the sensors based on woven and knitted CCF electrodes was first apparent at the pressure threshold of 14.3 kPa (Supplementary Materials Figure S1). The sensitivity of the knitted CCF-based pressure sensors was 1.5, 2, and 2.5 times greater than that of the woven CCF-based sensors at the applied pressure of 25.7 kPa, 40 kPa, and 100 kPa, respectively (Figure S1).

As shown in Figure 4d, the compression distance of the pressure sensor based on knitted CCF electrodes was greater than that of woven CCF electrodes under the applied pressure, thus supporting the higher sensitivity of woven CCF-based pressure sensors. Since the same dielectric layers were used, it is highly probable that the difference in pressure sensing performance between the two sensors stems from the inherent properties of the woven and knitted structures. Figure S2 shows that a knitted CCF is more compressible than a woven CCF under a given applied pressure. While a woven fabric is made by interlacing warps and wefts in a perpendicular direction, a knitted fabric is fabricated by interlooping courses and wales. The looping structure of a knitted CCF forms a space between the fabric and bulk Ecoflex, and between the adjacent wales (series of loops running lengthwise in a knitted fabric), making a knitted fabric bulkier than a woven fabric. This naturally formed space between the knitted electrodes and the dielectric is likely to function as a second dielectric layer in the capacitive pressure sensor, thereby inducing higher sensitivity than a woven structure [11].

### 3.4. Sensitivity Enhancement by Air-Gap Integration into a Porous Dielectric Layer

**Figure 5:**
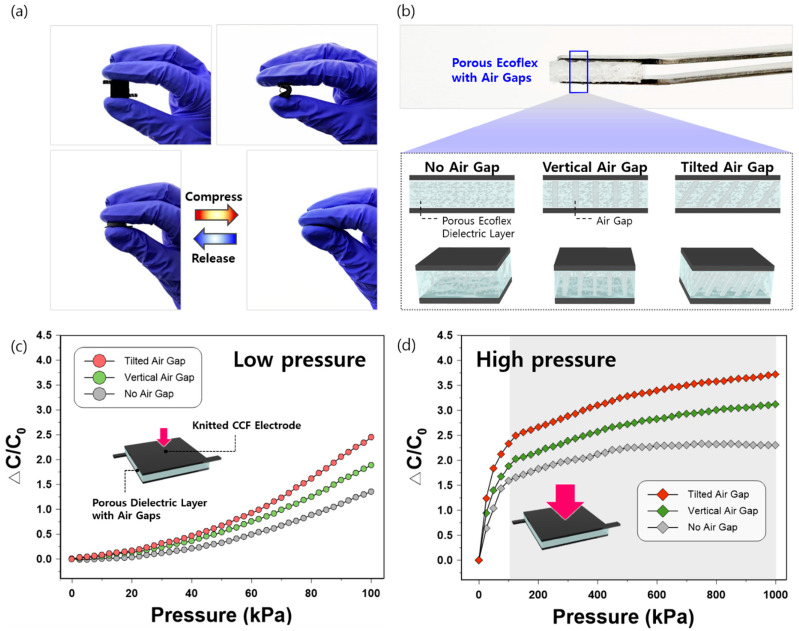
(**a**) Photographs of the CCF-based capacitive pressure sensor showing the mechanical flexibility and elasticity; (**b**) air gap structures of the capacitive pressure sensor within the porous dielectric layer; capacitance response of CCF-based pressure sensors based on the porous dielectric layer with no air gaps, vertical air gaps, and tilted air gaps at the applied pressure (**c**) from 0 to 100 kPa and (**d**) from 0 to 1000 kPa.

Given the advantages of the knitted structure in inducing higher sensitivity, we utilized knitted CCF electrodes in our sensor when structuring air gaps inside the porous dielectric layer to further enhance the sensitivity. As shown in Figure 5a, our sensor is highly flexible, elastic, and reversible. The air gaps in the porous dielectric layer were generated in two types: vertical and tilted structures (Figure 5b). The tilted air gaps in the structured elastomer demonstrated the highest pressure-sensing performance at an applied pressure of 100 kPa (Figure 5c). The sensitivities of pressure sensors with tilted, vertical, and no air gaps in the dielectric layers were 24.5 × 10^−3^, 18.8 × 10^−3^, and 13.5 × 10^−3^ kPa^−1^, respectively. The capacitance variations between the three air-gap structures showed greater differences as the applied pressure increased (100 kPa). The sensitivity of our best sensor based on a porous Ecoflex with tilted air gaps (24.5 × 10^−3^ kPa^−1^ at 100 kPa) was relatively high in comparison to that of sensors with a porous Ecoflex by the particle template method using sugar (12.1 × 10^−3^ kPa^−1^) [11] or salt (19.9 × 10^−3^ kPa^−1^) [12].

When a higher pressure of 1000 kPa was applied (Figure 5d), the sensitivity of capacitive pressure sensors with tilted, vertical, and no air gaps in the dielectric decreased to 3.7 × 10^−3^, 3.1 × 10^−3^, and 2.3 × 10^−3^ kPa^−1^, respectively. This is mainly due to the sharp decrease in capacitance changes observed at an applied pressure of 125 kPa (shaded in gray in Figure 5d). The decreasing tendency of sensor sensitivity observed in the three sensors in the higher pressure region (125–1000 kPa) can be attributed to the closure of structured pores in the dielectric layer and the elastic resistance of the remaining elastomer, as often addressed in previous research [13].

Based on the findings in Figure 5c,d, the following three features of the CCF-based sensor with tilted air gaps in the dielectric can be highlighted: (1) a wide detection range, (2) late saturation of the pressure-sensing performance, and (3) high sensitivity. First, the workable range of our sensor (0–1000 kPa) is outstanding compared to the sensing range of previous research on pressure sensors based on CCF. For example, Chang et al. [32] fabricated a flexible CCF/TPU pressure sensor whose detection range was limited to 0–16 kPa [32]. The multilayer pressure sensor based on CCF/PDMS [36] and the CCF sensor decorated with reduced graphene oxide [2] had a sensing range of 0–200 kPa and 0–500 kPa, respectively. The wide detection range of our sensor can be attributed to the porous structure of the dielectric between the CCF electrodes.

Second, the air gap structure in the dielectric may be advantageous in terms of delaying the pressure-sensing saturation of pressure sensors with a porous dielectric. While the structured pores in the dielectric can lead to wider operating ranges and higher sensitivity in capacitive pressure sensors by increasing the deformability [3,31,39], capacitive pressure sensors with porous dielectrics suffer from rapid saturation of the sensing performance [14,30]. It is worth noting that both sensors with an air gap structure (vertical and tilted) exhibited a steady increase in capacitance variations, whereas the pressure sensor with no air gap displayed saturated responses after an applied pressure of 500 kPa. This may show the advantages of air gaps that penetrate the dielectric layer; the air gaps can still contribute to the changes in the distance between CCF electrodes after the smaller pores become saturated, which may slow down the saturation of the capacitive pressure sensor.

**Figure 6:**
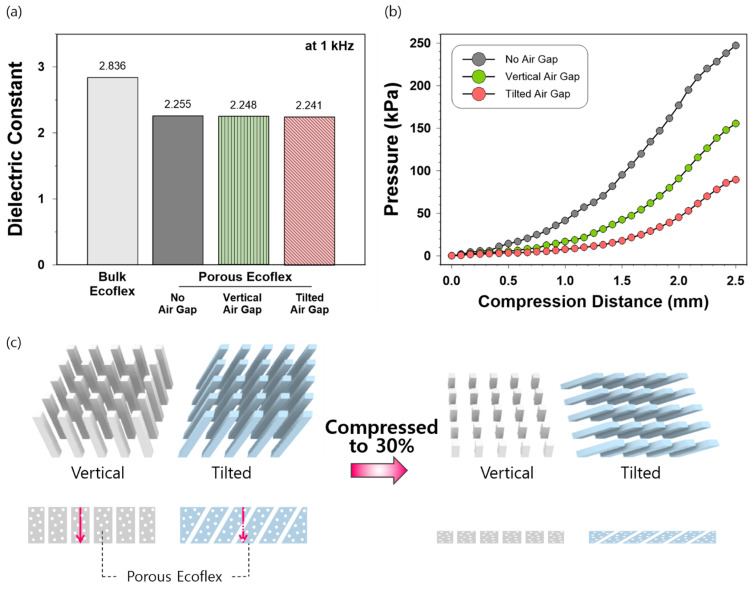
(**a**) Dielectric constants of the bulk dielectric, porous dielectric with no air gaps, vertical air gaps, and tilted air gaps at a frequency of 1 kHz; (**b**) relationship between the compression distance and applied pressure of CCF-based capacitive pressure sensors with no air gaps, vertical air gaps, and tilted air gaps; (**c**) schematic of air gaps before and after compression to 30%.

Finally, the sensor with tilted air gaps in the porous Ecoflex exhibited higher sensitivity than that with vertical air gaps in both low-and high-pressure regions. To understand why the tilted air gaps induced higher sensitivity when integrated into the dielectric of a pressure sensor, we compared the dielectric constants of the four different structures of elastomer, namely, bulk Ecoflex and porous Ecoflex with no air gaps, vertical air gaps, and tilted air gaps (Figure 6a). In addition, the compression distances of elastomers under the same applied pressure were explored (Figure 6b). The dielectric constant was measured using a Keysight E4980AL LCR meter with a dielectric material fixture (1020, Wayne Kerr, UK) using a non-contact electrode method at a frequency of 1 kHz. As shown in Figure 6a, the dielectric constants of the porous Ecoflex with and without air gaps were lower than those of bulk Ecoflex (no pores, no air gaps) owing to the increased number of voids. Notably, the dielectric constants of the porous Ecoflex with no, vertical, and tilted air gaps did not significantly differ, which indicates that the air gaps in the dielectric may not significantly affect the initial relative permittivity of a material.

However, as demonstrated in Figure 6b, the sensor with the porous dielectric with tilted air gaps exhibited the lowest level of applied pressure required to compress the sensor to a given distance. For example, the applied pressure to compress the sensors by 2 mm for the tilted gap structure was 45 kPa, whereas those for the vertical and no air gap structures were 91 and 177 kPa, respectively. Therefore, the higher deformability found in the porous Ecoflex with tilted air gaps is likely to result in greater changes in capacitance when compressed, thereby increasing the sensitivity. Figure 6c presents a schematic of the vertical and tilted air gaps before and after compression. In a vertical structure, air gaps are less likely to affect the compressibility of the adjacent part of the porous Ecoflex. However, the tilted gaps create empty spaces near the remaining Ecoflex; less force is required to compress the tilted structure owing to tilted air gaps that substitute the porous Ecoflex in the direction of pressure. Therefore, the tilted structure of air gaps may be more effective in reducing the stiffness of elastomer than the vertical structure. In addition, as observed in the 70% compressed state, the tilting angle of 60° decreases as pressure is applied, which may increase the space occupied by tilted air gaps in the dielectric.

Accordingly, it is probable that the higher sensitivity observed in the tilted air-gap structure was induced by higher deformability, which effectively increased the changes in distances between the electrodes, with less relation to the initial dielectric constants of materials. Here, it should be noted that our investigation does not consider the differences in the effective dielectric constant under compression stemming from structural difference, which deserves further systematic investigation. In addition, the tilting angels of the air gaps in the dielectric layer may significantly affect the pressure-sensing performance, such as sensitivity and working ranges. The optimum of tilting angles, which our study did not explore, should be demonstrated in future research. Furthermore, Luo et al. [23] fabricated a capacitive pressure sensor based on tilted micropillar arrays and attributed the sensitivity enhancement effect of the tilted structure to the bending deformation. They mentioned that bending deformation would facilitate the distance changes between the electrodes compared to compression deformation that traditional vertical arrays undergo. This phenomenon may be applied to the structure of our sensor, which should be further investigated in future research.

**Figure 7:**
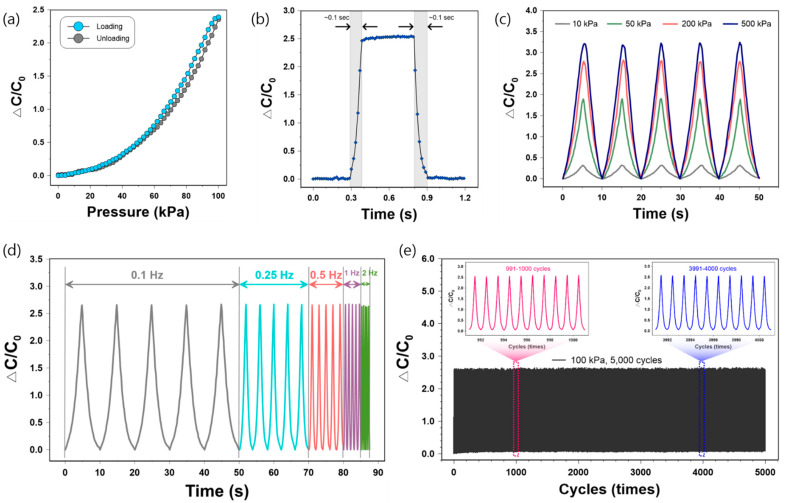
Typical pressure-sensing characteristics. (**a**) Hysteresis properties of CCF-based capacitive pressure sensors; (**b**) response and recovery time of pressure sensors; (**c**) dynamic response of pressure sensors under periodic applied pressures of 10, 50, 200, and 500 kPa; (**d**) dynamic response of pressure sensors at different frequencies of 0.1, 0.25, 0.5, 1, and 2 Hz; (**e**) capacitance response of pressure sensor during 5000 loading and unloading cycles at an applied pressure of 100 kPa.

The pressure-sensing properties of our best CCF-based capacitive pressure sensor (a porous dielectric layer with tilted air gaps) were characterized (Figure 7). As demonstrated in Figure 7a, our sensor exhibited reversible capacitance variations without notable hysteresis. The degree of hysteresis (*DH*), which quantitatively indicates the hysteresis performance, was calculated as follows (Equation (3)) [40]:

(3) $$DH=\frac{A_{Loading}-A_{Unloading}}{A_{Loading}}\times 100\;\left(\%\right)$$

where *A_Loading_* and *A_Unloading_* are the areas under the loading and unloading curves, respectively. The *DH* of the proposed CCF-based pressure sensor was 8.8%. This low hysteresis can be attributed to the existence of pores and air gaps, which decreases the volume fraction of Ecoflex. Therefore, the viscoelastic characteristics of Ecoflex are reduced in structured elastomers, inducing negligible hysteresis [41].

When a pressure of 100 kPa was applied, the capacitance of the CCF-based capacitive pressure sensor with tilted air gaps displayed immediate increase with a response time of less than 0.1 s (Figure 7b). When the pressure was released, the sensor also exhibited rapid recovery time of less than 0.1 s (Figure 7b). Figure 7c shows the capacitance responses of the sensor under various periodic pressures of 10, 50, 200, and 500 kPa at 0.1 Hz. The electrical responses of the sensor were highly reversible and stable during the five loading and unloading cycles, which can be attributed to the elastic properties of the Ecoflex dielectric layer. As already shown in Figure 5c,d, the signal amplitudes did not necessarily correspond to an increase in the applied pressure.

To evaluate the pressure-sensing responses of the pressure sensor at higher frequencies, we tested the sensor performance under an applied pressure of 100 kPa by increasing the frequency from 0.1 to 2 Hz (Figure 7d). Notably, the sensor signals at different frequencies displayed stable dynamic responses with no apparent changes in amplitude, except for at 2 Hz where the amplitude showed slight decreases. Durability is of great importance for the long-term practical application of pressure sensors, showing that it can maintain their performance after repeated use. Figure 7e presents the capacitance variations in the CCF-based pressure sensor with tilted air gaps under a pressure of 100 kPa during 5000 loading–unloading cycles. The performance of the pressure sensor was stable after 5000 cycles. This outstanding reproducibility and durability can be ascribed to the highly resilient characteristics of Ecoflex as a dielectric layer.

### 3.5. Applications of CCF-Based Capacitive Pressure Sensor in Human-Motion Monitoring

**Figure 8:**
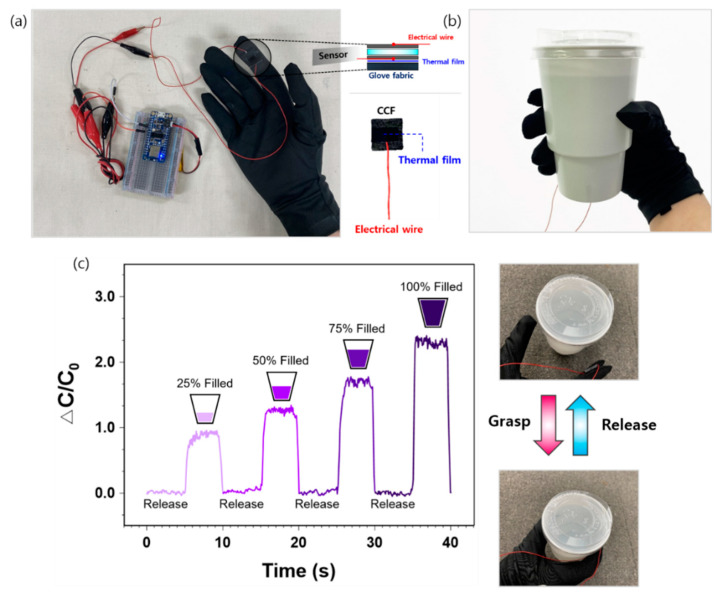
(**a**) Prototype of the finger-grasping-monitoring glove; (**b**) water-cup grasping with the smart glove; (**c**) capacitance variations of grasping motions with a 25%, 50%, 75%, and 100% filled water cup.

The wearable applications of the proposed capacitive pressure sensor using a porous Ecoflex with tilted air gaps as the dielectric layer and knitted CCF electrodes were tested through grasping motions. As shown in Figure 8a, we developed a smart glove using the sensor as a portable prototype. Electrical wires (AWG 32) were fixed on the top and bottom sides of the CCF electrode using a thin thermal film by a heat press (ISP0450MR, INNOSTA, Hanam, Korea) at 150 °C for 15 s to ensure a firm connection between the wires and electrodes. The sensor was integrated into the index finger of the glove using a double-sided thermal film and the same heat press. The prototype glove with the developed sensor was prepared by connecting it to a hardware platform. The hardware platform included an integrated microcontroller (MCU) with Bluetooth 5.0 (Nrf52840), a lipo-battery (3.7 V), and a resistor (2 MΩ). The sensing signal was collected every 50 ms and smoothed using a digital moving average filter (finite impulse response filter). It accepts 10 input samples at a time and takes their average to produce a single output point. The average value is transmitted to a phone, desktop, or tablet monitor using a wireless protocol (Bluetooth low-energy).

During the grasping test, subjects were asked to grasp and hold a water cup (200 g) for 10 s (Figure 8b), followed by a 10 s release. The sensor signal immediately increased upon grasping the cup and decreased during the release period (Figure 8c). The CCF-based capacitive pressure sensor was sensitive enough to detect the changes in cup weight, which was controlled by pouring 75, 150, 225, and 300 mL water to fill the cup by 25%, 50%, 75%, and 100%, respectively. The amplitude showed an apparent increase with the amount of water. This sensitive and repeatable pressure-sensing performance demonstrates the possible applications of the sensor to wearable electronics or smart robotics, where monitoring is required for grasping motions. However, the capacitance variations in the grasping motions showed slight noise during the 5-s grasping and 5-s releasing periods (Table S1), which should be addressed and considered for practical applications. In addition to robotic fingers, our sensor may also be applied to human-motion monitoring such as finger and knee bending, walking, running, and jumping. The scalable fabrication process of the proposed sensor may be particularly beneficial to these applications; the sensor can be readily fabricated by the straightforward process with inexpensive low materials (Ecoflex, sugar, and cotton).

## 4. Conclusions

In conclusion, we presented a novel capacitive pressure sensor with CCF electrodes and an air-gapped porous Ecoflex using an easy, low-cost, and scalable strategy. Commercial cotton fabric was converted into electrodes by thermal treatment at 800 °C, and the air-gapped porous Ecoflex was fabricated using sugar particles and a 5 × 5 array of metal pins. The combination of knitted CCF electrodes and the porous dielectric with tilted air gaps yielded the highest sensitivity (24.5 × 10^−3^ kPa^−1^ at 100 kPa). This can be attributed to the naturally formed space in knitted fabrics and the effectiveness of the tilted structure in reducing the stiffness of the dielectric, leading to its high deformability. In addition, the proposed sensor exhibited outstanding flexibility, reversibility, durability, low hysteresis, fast response and recovery time, and a wide detection range (1 MPa). Furthermore, because the electrodes of the sensor were derived from cotton, the sensor can be highly biocompatible and ecofriendly. To demonstrate its applications, we developed a prototype smart glove to monitor grasping motions. The sensor was sensitive enough to detect grasping motions under different cup weights. Therefore, we expect that this newly developed sensor with a facile yet effective strategy has great potential in a wide range of applications.

## Data Availability

Not applicable.

## Supplementary Materials

The following are available online at https://www.mdpi.com/article/10.3390/s21113895/s1, Figure S1: Sensitivity curves of capacitive pressure sensors with knitted and woven CCF electrodes. The blow arrow indicates the pressure threshold where the sensitivity differences appeared between the knitted and woven CCF-based pressure sensors. The purple dotted lines show the pressure levels where the sensitivity of the knitted CCF-based sensor was 1.5, 2, and 2.5 times greater than that of the woven CCF-based sensor, Figure S2: Relationship between the compression distance and applied pressure of knitted and woven carbonized cotton fabrics, Table S1: Variances of the capacitance variations of 5-s releasing and 5-s grasping motions with a 25%, 50%, 75%, and 100% filled water cup.
