# Supplementary material for: Carbonized Cotton Fabric-Based Flexible Capacitive Pressure Sensor Using a Porous Dielectric Layer with Tilted Air Gaps
Source: Sensors (Basel). 2021 Jun 4;21(11):3895. doi: 10.3390/s21113895 (PMC8200227; doi:10.3390/s21113895)
Supplement: Supplementary file 1 [file sensors-21-03895-s001.zip › sensors-1232996-supplementary.pdf]

Supplementary Materials for

Carbonized Cotton Fabric-based Flexible Capacitive Pressure Sensor Using a Porous Dielectric Layer with Tilted Air Gaps

Yelin Ko [1], Chi Cuong Vu [1], and Jooyong Kim [1,*]

[1] Department of Organic Materials and Fiber Engineering, Soongsil University, Seoul 06978, Korea

*Correspondence: jykim@ssu.ac.kr; Tel.: +82-2-820-0631

This PDF file includes:

Figure S1, Figure S2, and Table S1

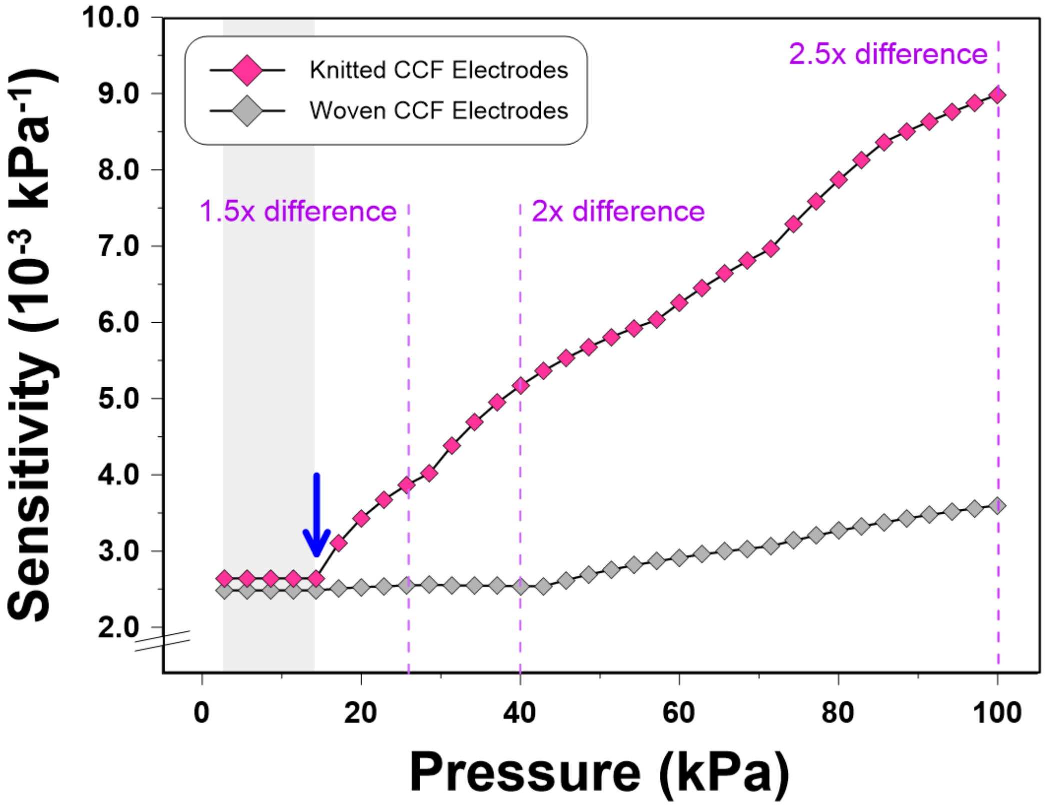

Figure S1. Sensitivity curves of capacitive pressure sensors with knitted and woven CCF electrodes. The blow arrow indicates the pressure threshold where the sensitivity differences appeared between the knitted and woven CCF-based pressure sensors. The purple dotted lines show the pressure levels where the sensitivity of the knitted CCF-based sensor was 1.5, 2, and 2.5 times greater than that of the woven CCF-based sensor.

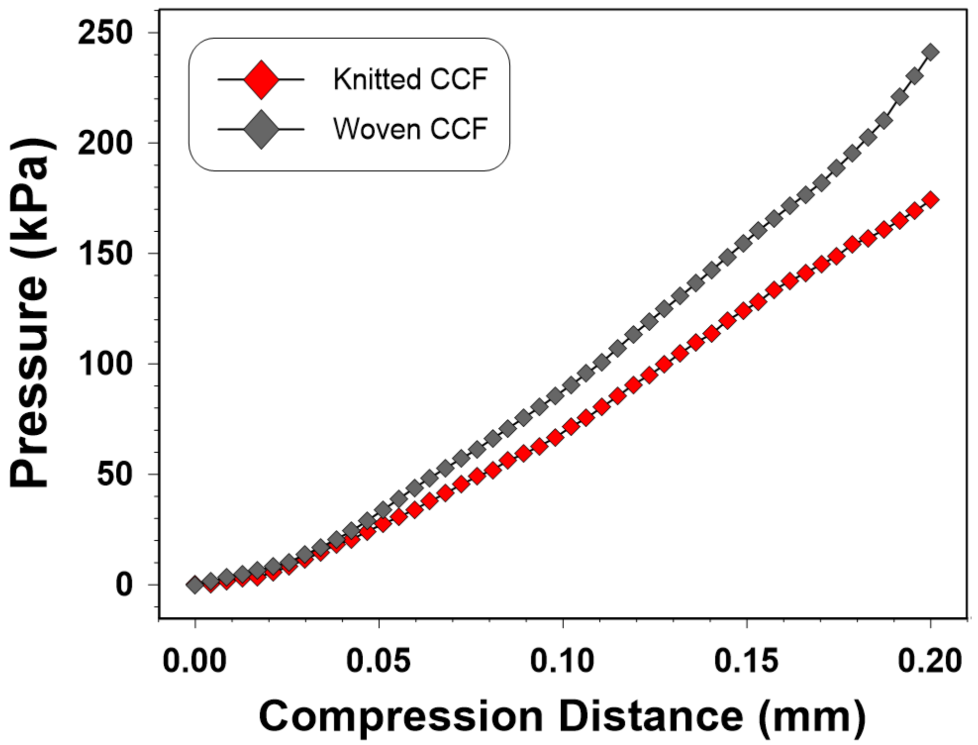

Figure S2. Relationship between the compression distance and applied pressure of knitted and woven carbonized cotton fabrics.

Table S1. Variances of the capacitance variations of 5-s releasing and 5-s grasping motions with a 25%, 50%, 75%, and 100% filled water cup.

Motion	25% Filled	50% Filled	75% Filled	100% Filled
Release	0.00034	0.00054	0.00087	0.00083
Grasp	0.04956	0.07790	0.21745	0.30475
